# Improving depressive symptoms and maintaining cognitive abilities of seniors within the nursing homes: A pilot study of brief mindfulness-based interventions for seniors in a semi-randomized trial

**DOI:** 10.3389/fpsyg.2022.994336

**Published:** 2023-08-09

**Authors:** Daniela Aisenberg-Shafran, Margalit Harmatz

**Affiliations:** Clinical Psychology of Adulthood and Aging, Ruppin Academic Center, Emek Hefer, Israel

**Keywords:** mindfulness, cognitive control, emotional distress, depression, nursing homes

## Abstract

**Introduction:**

Seniors in nursing homes are at higher risk for depression and emotional distress. The COVID-19 crisis and isolation have even increased the risk for cognitive decline and suicidality. Since social media is often unfamiliar to older adults, their treatment options were diminished during quarantine. Recently, studies showed the potential for Mindfulness-Based Intervention in improving cognitive functioning and psychological well-being among healthy older adults. Standardized courses such as MBSR and MBCT are not suited to the majority of seniors for several reasons. First, the interventions are too long and demanding, physically and cognitively. Second, they require an instructed counselor for delivery, which makes it almost impossible in times of quarantine, and third, very expensive. Hence, the purpose of this study was to examine whether similar improvements in emotional distress and cognitive functioning can be achieved through a brief intervention, that can be delivered by workers in nursing homes.

**Methods:**

A course of 8 half-hour sessions each (MBIS: Mindfulness-Based Intervention for Seniors) was employed in two versions: (1) An 8-week course with weekly meetings (2) A 4-week course with 2 sessions per week and compared to a control care-as-usual group. Depression and mood were measured, as well as cognitive abilities in the Simon task. In addition, the level of Mindfulness skills was measured before and after the interventions.

**Results:**

We showed that brief interventions succeeded in improving mindfulness *Non-reactivity* and produced changes in the facets *Acting with awareness* and *Non-judging*. More importantly, the brief intervention, in both frequency versions, improved the level of depression and mood (BDI and PHQ-9). At the cognitive level, an adaptive sequential effect appeared after the intervention only in the 8-week MBIS group.

**Discussion:**

These findings indicate the effectiveness of this pilot of a short, simple, mindfulness-based intervention, in improving depression and psychological distress, as well as improving cognitive control over time. This may enhance significant developments in the field of treatment solutions for seniors, with a ready-to-use protocol to administer in nursing homes.

## Introduction

The process of aging involves many challenges, more than at any other stage in life. The aging individual is exposed to deaths, separations, and a decline in emotional and cognitive functioning. The COVID-19 crisis enforced the isolation of seniors, putting them at higher risk for depression and emotional distress (Armitage and Nellums, 2020). Since social media is often unfamiliar to the older adults, their treatment options were diminished during quarantine. This situation increased age-related effects and even cognitive changes. The exercise of Mindfulness has been found to improve psychological wellbeing and other cognitive measures among both young individuals and seniors. Standard mindfulness-based interventions (MBI) are lengthy and demanding and are largely unsuitable for seniors. This research suggests a pilot study of a brief mindfulness-based intervention appropriate for seniors (Mindfulness Based Intervention for Seniors: MBIS), for administration in nursing homes or retirement communities.

Emotional distress and depression in old age are influenced by a complex combination of factors (Lomas et al., 2016). The decline in mobility, the increase in social isolation, the increase of physical and cognitive disease, and the loss of close relationships increase the risk for depression (Rodda et al., 2011). There is an international increase in the percentage of individuals suffering from depression. One of the reasons for this increase in depression is the increase in longevity (World Health Organization, 2017), as more than half of those suffering from depression in old age have experienced their first outbreak late in life. Some results of depression include a decrease in life satisfaction, avoidance of social relationships, isolation, an increased need for 24/7 care, cognitive decline, disability in daily functioning, and suicide (Fiske et al., 2009). Emotional distress and cognitive decline have a strong mutual influence on one another in old age. Depression and distress accelerate cognitive decline among those aging (Engmann, 2011), while cognitive decline influences the emotional state and is very damaging to the quality of life of the aging individual (Park et al., 2003).

Cognitive control is the brain’s capacity to actively create information that will guide behavior in accordance with a set goal (Posner and Snyder, 1975). This is the process that allows an individual to choose a certain behavior and reject an inappropriate behavior. The ability to delay or inhibit responding is one of the necessary features of cognitive control. It was found that the performance efficacy of tasks that require cognitive and inhibitory control declines with age (Hasher and Zacks, 1988; West and Alain, 2000; Maylor et al., 2011; Diamond, 2013). A task that requires cognitive and inhibitory control, and is often used in research on seniors is the Simon task (Simon and Small, 1969; Bialystok et al., 2004; Proctor et al., 2005; Castel et al., 2007; Germain and Collette, 2008; Juncos-Rabadán et al., 2008; Kubo-Kawai and Kawai, 2010; Tse et al., 2010; Maylor et al., 2011). In the Visual Simon task (Craft and Simon, 1970; Proctor and Lu, 1994), the participant is requested to identify colored circles that appear on a computer screen and press the appropriate key, on the left or right side. In this task, the colored circle can appear on the same side on which the participant is supposed to press the key (congruent condition) or on the opposite side (incongruent condition). The Simon effect is defined as the gap in response times (RTs) between the congruent and incongruent conditions. Research has shown that seniors have longer RTs and a larger Simon effect when compared to younger adults (Van der Lubbe and Verleger, 2002; Aisenberg et al., 2014). The Simon effect among seniors is significant even after a statistical correction that considers the generally slow motor functioning associated with aging processes (Van der Lubbe and Verleger, 2002). Aisenberg et al. (2014) compared the performance of senior participants and young participants in the Simon task, using neutral trials (a colored circle in the middle, above or below), as well, and found that the Simon effect is greater among senior participants, due to a difficulty performing incongruent trials. When examining sequential effects, which relate to the influence of the previous trial on the performance in the current trial, the Gratton effect was found among young participants (Gratton et al., 1992). The Gratton effect is the decline that appears in the Simon effect after the response to an incongruent condition, rather than after the response to a congruent condition (Wühr and Ansorge, 2005; Aisenberg and Henik, 2012). Botvinick et al. (2001) suggested that this sequential effect reflects the functioning of dynamic cognitive control of the reactive type. Aisenberg et al. (2014) found that the Gratton effect does not appear in senior participants, reflecting damage to the dynamic mechanism of inhibited responding. There is, however, a debate in the literature regarding the significance of the effect (e.g., see the explanation of repetition priming in Maylor et al., 2011).

As long as the population ages, there is great importance in developing interventions that can influence the psychological distress and cognitive functioning of seniors. In this study, we chose to examine the influence of a mindfulness-based intervention on psychological well-being and cognitive capacity in old age.

The beneficial influence of mindfulness exercises on psychological and physical health has been known in the East for thousands of years. Jon Kabat-Zinn defined Mindfulness as, “The awareness that arises through paying attention, on purpose, in the present moment, non-judgementally” (Kabat-Zinn, 1994, p. 4). He developed the Mindfulness-Based Stress Reduction program (MBSR), intended to treat stress and pain (Kabat-Zinn, 1994). Based on this program, Mindfulness-Based Cognitive Therapy (MBCT) was developed to treat depression (Segal et al., 2012). A plethora of mindfulness-based intervention courses were developed to treat different issues (Eating Awareness—Kristeller et al., 2006; Elder Care—Mcbee, 2008). The goal of these courses is to develop and cultivate mindfulness in meditation and in day-to-day life. This is done *via* the practice of non-judgmental attention to internal events (i.e., breathing, body sensation, emotions, and thoughts) and external events (i.e., sound, smell, taste, and touch). Such practice is accompanied by psychoeducation. Course participants are also asked to exercise two types of practice every day at home: formal practice and informal practice. The formal practice includes a repetition of the exercise done at the course session, and the informal practice includes daily behaviors of mindful attention. One of the most popular questionnaires for measuring mindfulness is the Five Facets Mindfulness Questionnaire (FFMQ), developed by Baer et al. (2006). This questionnaire is a self-report questionnaire, and it measures the five facets of mindfulness: Observing; Describing; Acting with awareness; Nonjudging of inner experience; and Nonreactivity to inner experience (elaboration of this tool is in the methods section of this paper).

In seniors, MBI interventions have consistently been shown to improve emotional distress in seniors (Geiger et al., 2016). A significant influence was found for anxiety, depression, and stress—a similar influence to that found in young, healthy participants (Khoury et al., 2015). Ernst et al. (2008) reported an improvement in quality of life and depression as a result of participation in an appropriate MBSR program, when compared to senior participants on the waiting list. The positive influence of meditation on attention has been found in both, cross-sectional and longitudinal studies. In research that compared the attention of senior participants that have practiced meditation for years with the attention of senior participants that have never practiced meditation, there was a significant difference between the groups (Van Leeuwen et al., 2009). In addition, Moynihan et al. (2013) examined the influence of meditation on executive functioning after participation in an MBSR course and found a significant improvement in executive functioning (Trails B/A ratio). Sun et al. (2013) found that seniors that completed a course on meditation improved in their performance of the Forward Digit Span (though, not in their performance of the Backward Digit Span), which can point to an improvement in working memory.

Gard et al. (2014) analyzed research studies that examined the influence of different meditation techniques on the cognitive functions of senior participants and found many limitations. Most of the studies were preliminary pilot studies and included a small number of participants. Some of the studies did not include a control group that participated in a different intervention. Another limitation was that the different meditation interventions were not standardized, which makes it difficult to draw clear conclusions from the group of studies that were analyzed. The researchers concluded that the preliminary results reflect the potential of meditation on the delay of cognitive decline among seniors. The researchers simultaneously emphasized the need for further research on the topic.

A varied range of results regarding MBI’s capacity to improve mindfulness in seniors has been found. Morone et al. (2009) did not see any change in mindfulness levels in senior participants that suffered from chronic back pain and participated in an MBSR course. Also, Mularski et al. (2009) did not find a difference in mindfulness levels between the group of senior MBI participants and the control group, after the intervention. Both these research studies hypothesized that the reason for no apparent change is due to the fact that the average score of participants on the mindfulness questionnaire prior to participation in the course was particularly high. In contrast, Lenze et al. (2014) found an improvement in mindfulness levels among senior participants after their participation in the MBSR course. These studies made use of self-report questionnaires.

The need for brief Mindfulness courses arises since the standard MBI course (e.g., MBSR and MBCT) that lasts for a duration of 8 weeks, includes 2 3-h sessions, a retreat, which lasts 6 h, and a requirement to complete 40 min of daily practice at home. Research has shown that the main reason for dropout in Mindfulness-Based Meditation courses (MBCT and MBSR) is the amount of time they require of the participants (Carmody et al., 2008). This has led to an exploration of different ways of abbreviating the MBI interventions. Shortening interventions is especially important in the senior population, as sessions that last about 2 hours or that require practice for more than half an hour at home, are not appropriate for a majority of individuals in this age group (Mcbee, 2014; Helmes and Ward, 2017).

In a meta-analysis conducted by Carmody and Baer (2009), for young adults, they did not find support that shorter versions of MBSR sessions are less efficacious than the standard format (2 ½ hours) in decreasing psychological distress. Klatt et al. (2009) found that after an MBSR intervention that was 6 weeks long and included weekly sessions of an hour (total class-time: 6 h + 20 min a day of practice at home), there was an improvement in mindfulness and psychological distress. Tang et al. (2007) reported an improvement in attention management, mood, and physiological measures of stress, after participation in a short meditation course intended for young participants. The course only included 2.5 h of class-time. The control condition in this research participated in a relaxation course with a similar time allotment. Attention management was measured *via* the Attention Network Test (ANT; Fan et al., 2002). Change in mood was measured *via* the Profile of Mood States questionnaire (POMS; Shacham, 1983). This course included a special set of exercises, which included body relaxation, guided imagery, and mindfulness meditation with background music. The course did not include any instruction to rid the mind of thoughts, as the researchers believed this instruction to be too difficult for inexperienced meditators to implement. There have also been attempts at appropriating MBSR courses for the senior population. Such courses have shown improvement in psychological and cognitive measures. However, the appropriated courses were still lengthy and demanding and, largely, inappropriate for the senior population. For example, they included weekly 2-h sessions, instead of 2 ½ hours, with no retreat (Ernst et al., 2008), or a weekly session of a 1 ½ hour instead of 2 ½ hours, with no retreat (Foulk et al., 2014).

The aim of the present study was to examine whether a brief mindfulness-based intervention (eight 30-min sessions), delivered in nursing homes, improves measures of emotional distress and cognitive control in seniors. Thus, the Mindfulness-Based Intervention for Seniors (MBIS) course was established (see Description in the methods section of this paper). In addition, and in light of findings by Tang et al. (2007) that showed improvement in a concentrated course, we examined the influence of session frequency on the efficacy of the course. Therefore, the MBIS was offered in two versions which differed in session frequency though maintained an equivalent total class-time of 4 h:

Version A: An 8-week course, one session per week (MBIS-1*8).
Version B: A 4-week course, two sessions per week (MBIS-2*4).
MBIS groups were compared to a third care-as-usual control group who had weekly gatherings following a joint breakfast, with no programmed intervention.

We hypothesized:

1. A main effect of time in all three groups for the reaction time in the cognitive task (repeating the task effect).
2. A significant difference in cognitive and emotional effects between both MBIS groups and the control group. Considering that participants in this study are seniors, time alone is not expected to have a positive influence on cognitive abilities and emotional distress. Test re-test effects will be examined using the control group, without experimental manipulation.

Within the MBIS groups:

3. Significant effect of time for:

3.1. Mindfulness measures, such that the interventions will encourage improvement in all measures of mindfulness.

3.2. Emotional distress, such that the interventions will improve measures of mood and depression.

3.3. Cognitive control. The measure expected to change is the Simon effect, such that the slowed response time in the incongruent condition will decrease as a result of the intervention. Likewise, and in accordance with Colzato et al. (2015) findings, we hypothesized that there will be a Gratton type sequential effect, following the interventions.

4. An interaction between time and group in the emotional measures, due to the change in the length and frequency of the sessions. We did not hypothesize a specific direction, as the high frequency in the MBIS 2*4 course can be exciting and encourage a fast change, while the lengthier duration of the MBIS 1*8 course can encourage a more stable improvement in one’s emotional experience.

## Materials and methods

The study received IRB approval from the ethics committee of the School of Social and Community Sciences at Ruppin Academic Center (pre-registration: clinicaltrials, NCT04165005, 15/11/19). The courses were conducted during November and December 2017, before pre-registration was a restriction, so the study registered as part of a bigger project in 2019, declaring that the data was already collected.

### Participants

Power analysis was made using G*Power (3: Faul et al., 2007), yielding *N* of 65, which were invited to participate. Following the recruitment process, 35 healthy seniors (31 women; *M = 80*.*9*, *SD = 3*.*9*; Range = 72–91) were administered from four assisted living facilities in Israel. Out of these, 19 were semi-randomly allocated to the MBIS course in one of its two versions (MBIS-2*4 or MBIS-1*8). All participants met with an experimenter, underwent a Mini Mental State Examination (MMSE; Folstein et al., 1975), a cognitive control task (Simon task), and answered a few self-report questionnaires to assess measures of emotional distress (see below the tools section). All participants received a score higher than 24 on the MMSE. Eleven participants began the MBIS-2*4 course, and one left after three sessions (9% dropout rate). Eight participants began the MBIS-1*8 course, and three left after two sessions (38% dropout rate). Sixteen participants were administered to the control group. Reasons for dropout were: Difficulty following instructions due to hearing issues, decline in health, and the illness of a partner and a resulting difficulty in commitment. Four participants did not complete the courses (see below the design section). Hence, data from 31 participants were available for the pre-post analysis.

### Tools

Mindfulness-Based Intervention for Seniors (MBIS) course: A course based on the MBCT and MBSR courses was established. The course program was created according to the principles of model of Hölzel et al. (2011). The course included eight sessions of 30 min each. Each session included an exercise (10–20 min), psycho-education (5–10 min), and/or experience sharing (3–10 min). In addition, the course participants were requested to practice 10–20 min a day, in accordance with what they exercised in the session. Aside from the formal exercise, the course participants were also encouraged to integrate mindfulness tasks into their day-to-day lives. According to model of Hölzel et al. (2011), the course began with Attention Regulation and Body Awareness exercises. With time, the exercises that activate Emotion Regulation and Change in Perspective on the Self processes were added. The psycho-education and sharing emphasized the relevance and significance of life for the participants.

Mindfulness Skills were measured *via* the Five Facets Mindfulness Questionnaire (FFMQ; Baer et al., 2006; In Hebrew: Tarrasch and Berger, 2022). This questionnaire is a 36-item self-report questionnaire, with five sub-scales: Observing; Describing; Acting with awareness; Non-judging of inner experience; and Non-reactivity to inner experience. Responses are reported on a five-point Likert scale, ranging from “Never or with very little probability” to “Almost always or usually correct,” the latter reflecting a high level of mindfulness. This questionnaire has also been shown to have high reliability and validity.

Emotional distress was measured via Hebrew self-report questionnaires for depression (BDI-II) and mood (PHQ-9), validated by the Israeli ministry of health. The BDI-II is an updated version of Beck’s depression questionnaire (Beck et al., 1996). It is a 21-item multiple-choice questionnaire and is validated and reliable for self-reporting. Each item has four answer options, each receiving 0–3 points. The higher the total score, the higher the depression level. The PHQ-9 is a sub-questionnaire of the PHQ-SADS questionnaire (Patient Health Questionnaire—Somatization, Anxiety, and Depression; Kroenke et al., 2010), which is used as a quick assessment of depression and mood.

Cognitive control was examined *via* the Simon task. The Simon task is particularly appropriate for senior participants as it has simple instructions and does not require language fluency. Stimulus presentation and data collection are done with laptops. Circles presented at a diameter of five degrees (a viewing distance of 60 cm from the screen) and colored blue, red, yellow, and green were used as the stimuli and were presented at one of four locations: left, right, above, or below the center of the screen.

**Figure 1:**
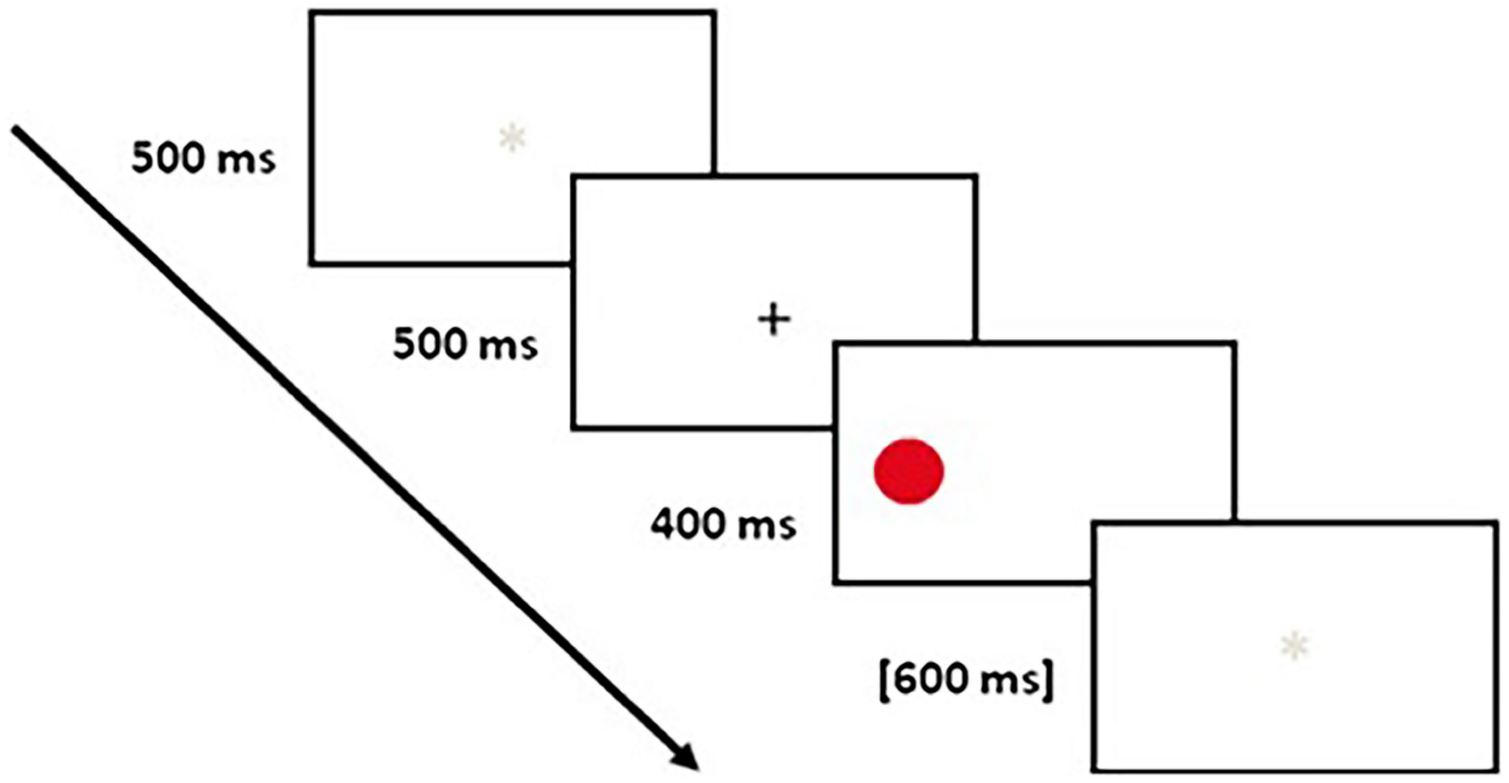
The four stages at each trial of the Simon task.

Choosing four colors, instead of two, was intended to negate the possibility of repetition priming (Maylor et al., 2011). Each trial included a white screen that appeared for 500 ms, after which a white screen with a black cross at the center was presented for 500 ms. Then, a stimulus appeared on the left, right, above, or below the center of the screen for 400 ms. After the stimulus disappeared, the white screen was presented for 600 ms (see Figure 1). The participants were requested to identify the colored circles that appeared on the screen and to press the appropriate button on the left or right side of the keyboard. They were requested to answer as quickly and as accurately as possible. First, the participants practiced 20 trials. Then, they performed the two parts of the experiment, each of which included 91 trials. The participants were permitted to take a break between the two parts of the experiment. For some of the participants, the “D” key represented red and green and the “L” key represented blue and yellow. For the other half of the participants, the key-color combination was reversed.

## Results

### Demographic parameters

A one-way ANOVA was performed to compare age, level of education, and MMSE score between groups, and a χ^2^ test was performed to compare gender (see Table 1). There were no differences between the groups in MMSE score and gender. *Post hoc* analyses using the Scheffé *post hoc* criterion for significance revealed age and level of education effects: the control group was significantly younger than the MBIS-2*4 group and the level of education of the MBIS-1*8 group was significantly higher than that of the other two groups.

**Table 1:** Averages (SDs) and differences and effects between the groups in the demographic and self-report variables.

	MBIS-1*8(*N* = 5)	MBIS-2*4(*N* = 10)	Control(*N* = 16)	All participants(*N* = 31)	Statistic parameter	*p<*	*η^2^_p_*
No. of women	4	10	12	26	*χ2* = 2.9	ns	
Age (yrs)	80.6 (2.7)	83.1 (4.4)^(+)^	79.1 (2.5)^(−)^	80.6 (3.7)	*F* (*1*,*28*) = 4.7	0.05	
Education (yrs)	17.2 (3.1)^(+)^	12 (2.5)^(−)^	12.1 (2.8)^(−)^	12.9 (3.3)	*F* (*1*,*28*) = −3.52	0.01	
MMSE	29.2 (1.9)	27.8 (2.9)	29 (1.0)	28.6 (2.0)	*F* (*1*,*28*) = 1.43	ns	
MMSE Before	29.2 (1.8)	27.8 (2.9)	29 (1.0)	28.6 (2.0)	All *Fs* (1,28) <1	ns	
MMSE After	29.0 (1.4)	28.5 (2.4)	28.8 (1.6)	28.7 (2.1)			
PHQ-9 Before	5.6 (2.7)	9.5 (5.3)	7.13 (3.3)	8.2 (4.8)	*F* (1,28) = 6.17	0.05	0.31
					For MBIS groups:		
PHQ-9 After	3.2 (0.8)	7.2 (5)	7.31 (2.4)	5.9 (4.5)	*F* = 11.73	0.01	0.29
BDI-II (MBIS-groups) Before	8.6 (3.8)	9.7 (6.9)	–	9.3 (5.9)^1^	*F* (1,13) = 3.3	0.09	0.2
BDI-II (MBIS-groups) After	6.8 (4.6)	7.8 (4.8)	–	7.5 (4.6)^1^			

(+/−) represents post-hoc significant differences between the groups; ^1^*N* = 15.

### Mindfulness measures (FFMQ)

**Figure 2:**
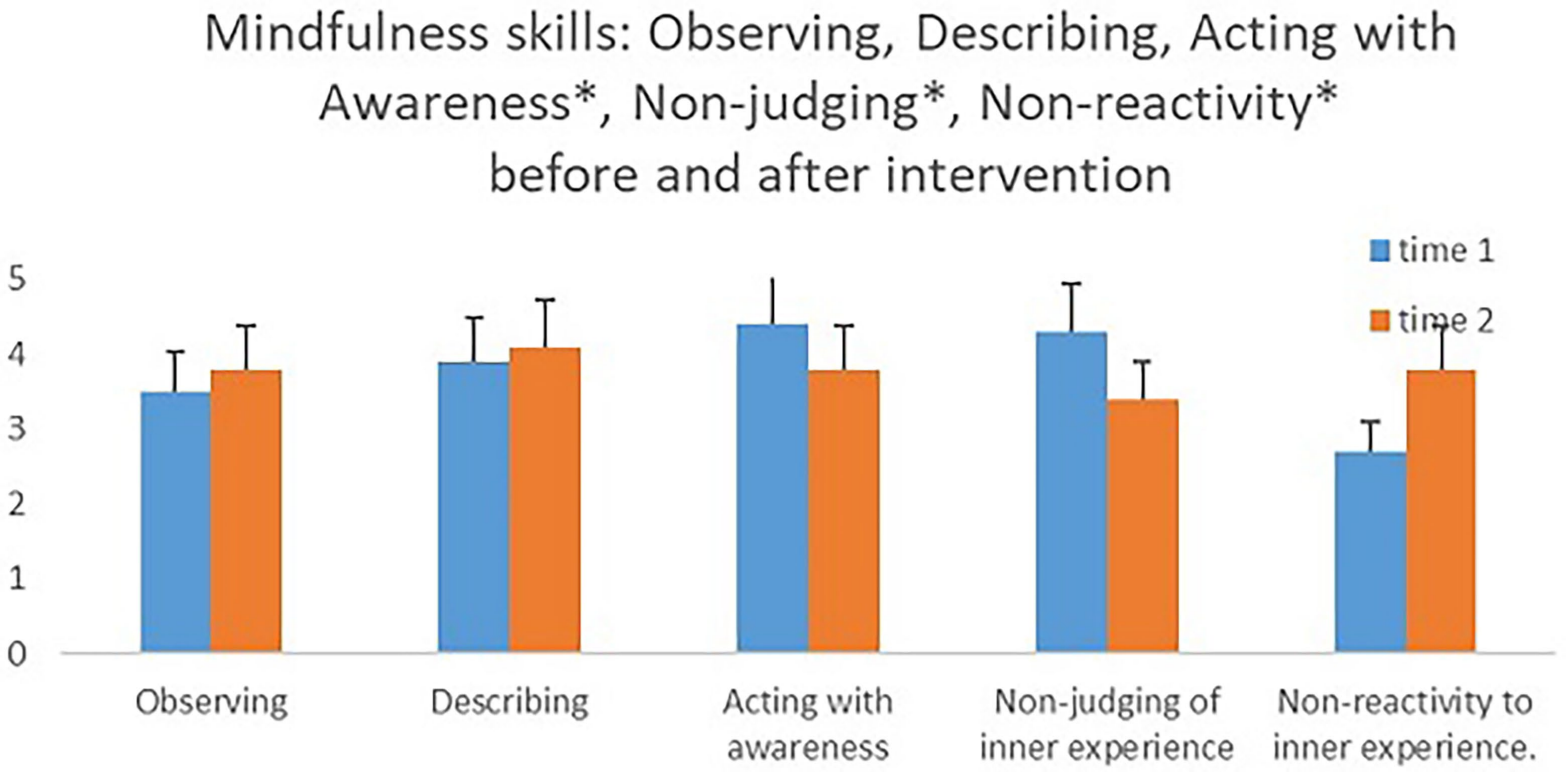
Mindfulness skills before and after the intervention.

In an attempt to examine if there were differences in mindfulness measures between the groups prior to the intervention, we conducted an independent samples *t*-test. There were no significant differences between the groups in any of the measures. A mixed model ANOVA test with within- and between-participant variables was conducted to examine the mindfulness measures as time-dependent (before and after the intervention), with course type (MBIS-2*4/MBIS-1*8) as a between-participant variable. The hypothesis that the different interventions will encourage an increase in mindfulness measures was only supported for the sub-scale, nonreactivity to inner experience. A significant main effect for time was found for this measure [*F*(1,12) *= 5*.*29*, *p < 0*.*05*, *η^2^ = 0*.*31*], such that its’ level after the intervention was higher (*M = 3*.*41*, *SD = 0*.*81*) than its level prior to the intervention (*M = 2*.*67*, *SD = 0*.*64*). In contrast, a significant main effect for time was found for the two sub-scales, acting with awareness and nonjudging of inner experience, though in the opposite direction from that which was hypothesized [*F*(*1*,*12*) *= 6*.*29*, *p < 0*.*05*, *η^2^ = 0*.*35*; *F*(*1*,*12*) *= 5*.*23*, *p < 0*.*05*, *η^2^ = 0*.*30*, respectively]; a decline in acting with awareness and a decline in nonjudging of inner experience after the intervention. No significant changes were apparent in the other mindfulness measures when their levels before and after the intervention were compared (see Figure 2 and Supplementary Table S1 in the Appendix).

### Emotional distress measures (PHQ-9, BDI-II)

**Figure 3:**
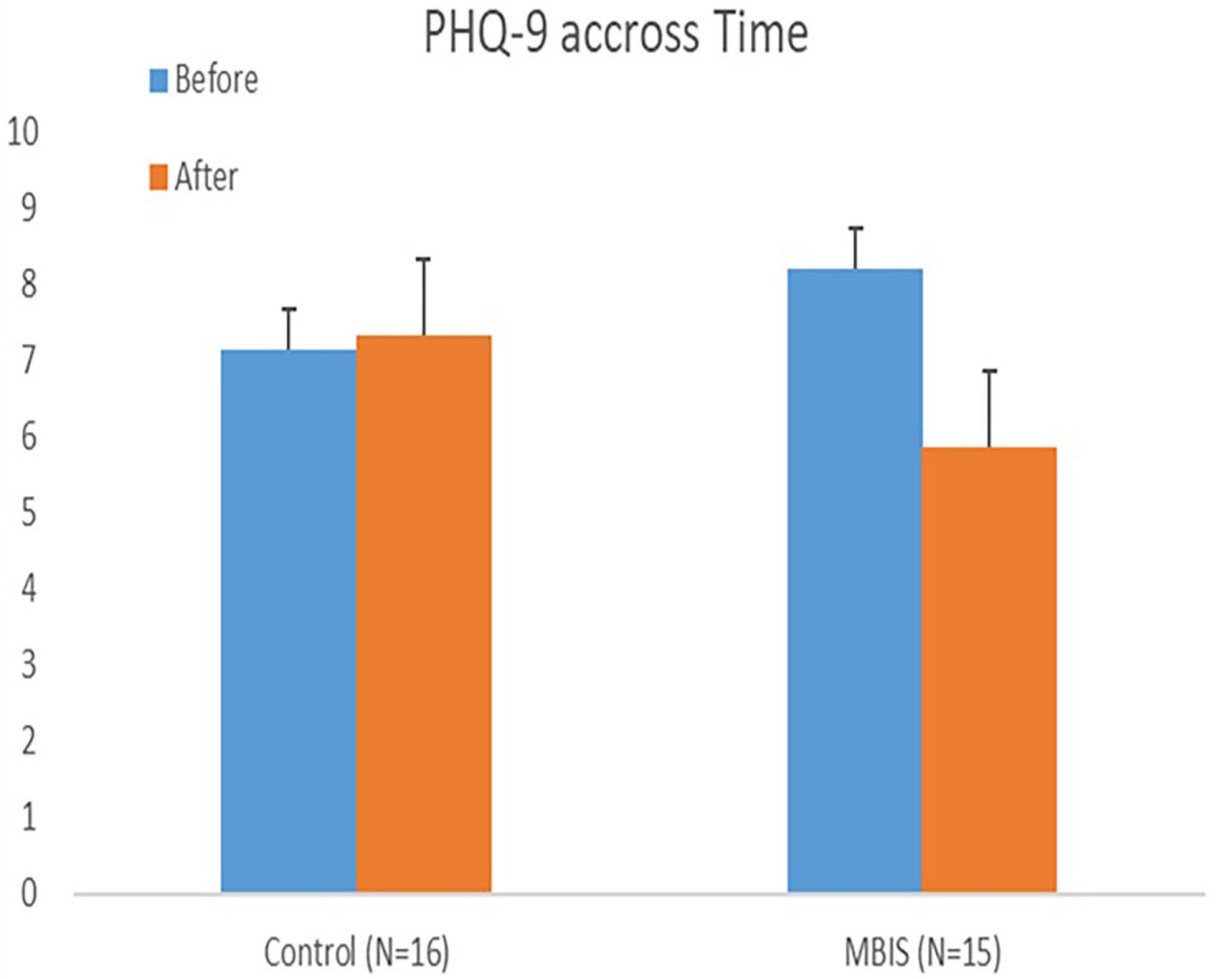
Depression level and mood before and after the intervention.

A mixed model ANOVA test with within- and between-participant variables was conducted to examine the emotional distress measures as time-dependent, with group type (MBIS-2*4/MBIS-1*8/control) as a between-participant variable. In accordance with the research hypothesis, a significant interaction effect of time-group was found in the PHQ-9 (mood) measure, such that this measure decreased as a result of the intervention (see Table 1). In other words, participants from both MBIS groups reported a more positive mood after the intervention (see Figure 3). Further, looking at the BDI-II measure of depression, tested for the MBIS groups but not the control group, a marginally significant main effect of time was found with an effect size of 0.2 (partial eta squared) and *F* greater than 3 (see Table 1), such that the reported level of depression after the intervention was lower than the reported level of depression prior to it. In contrast to our research hypothesis, there was no interaction between time and course type in any of the emotional distress measures (i.e., all *Fs < 1*).

### Cognitive functioning (MMSE, Simon task)

We conducted a mixed model ANOVA with within and between participant variables test to measure general cognitive functioning (MMSE) as dependent on time (before and after the intervention), with group type as a between participants (MBIS-2*4/MBIS-1*8/control) variable. Since we had a small sample size, a Shapiro–Wilk test was performed and did not show evidence of non-normality (*W = 0*.*92*, value of *p = 0*.*49*). Based on this outcome, and since the within-facility assignment was random, we decided to use a parametric test. Assumptions of the repeated measures ANOVAs were met with no violations [*Mauchly’s Test- χ^2^*(*2*) *= 2*.*042*, *p = 0*.*36*]. No main effect of time or group was found, and no time-group interaction was found either (see Table 1).

The average response time (RT) on the Simon task for each participant in each condition was calculated as the average of correct responses only. RTs that deviated more than 2.5 SDs from the average or that were lower than 150 ms were removed from the analysis. Further, one of the MBIS-2*4 participant’s data was removed from the final analysis due to a technical issue (see Table 2).

**Table 2:** Averages (SDs) of accuracy percentages in the Simon task and main effect for congruency.

	Before (Time 1)				After (Time 2)				*df*	*F*	*p*<	*η*^2^_p_
	MBIS-1*8 (*N* = 5)	MBIS-2*4 (*N* = 10)	Control (*N* = 16)	All participants (*N* = 31)	MBIS-1*8 (*N* = 5)	MBIS-2*4 (*N* = 10)	Control (*N* = 16)	All participants (*N* = 31)				
Congruent	93% (7%)	80% (0.35%)	94% (5%)	89% (17%)	96% (4%)	86% (6%)	95% (5%)	93% (8%)	2,52	11.70	0.000	0.31
Incongruent	89% (14%)	78% (36%)	92% (5%)	86% (15%)	95% (2%)	79% (23%)	91% (7%)	88% (11%)				
Neutral	96% (4%)	79% (6%)	94% (2%)	90% (19%)	98% (5%)	83% (2%)	97% (19%)	93% (10%)				

A mixed model ANOVA test with within- and between-participant variables was conducted to measure accuracy percentage for each group (MBIS-2*4/MBIS-1*8/control), time (before/after the intervention), and congruency (Congruent/Incongruent/neutral condition). The general accuracy percentage was high: 89% before the intervention; and 93% after the intervention. A main effect for congruency was found [*F*(*2*,*52*) *= 11*.*701*, *p < 0*.*00006*, *η^2^ = 0*.*31*], showing low accuracy rates for incongruent trials compared to congruent and neutral trials [*F*(*1*,*26*) *= 6*.*88*, *p < 0*.*014*, *η^2^ = 0*.*21*]. No other effects for accuracy were found (see Table 3).

**Table 3:** RT effects (RT in ms) in the Simon task.

**Effects**	**Meaning**	***df***	***F***	***p<***	***η***^***2***^_***p***_
Time	Trials on time1 faster than trials ontime2	1,26	8.3	0.007	0.24
Congruency	Congruent faster than Incongruent	1,26	25.3	0	0.49
Time × Congruency	Simon effect ontime2 larger Simon effect ontime1	1,26	4.6	0.05	0.15
Group × Congruency		2,26	4.78	0.017	0.27
Planned Comparisons	Simon effect for MBIS -1*8 is the smallest	1,26	7.19	0.012	0.22
Prev-Congruency	Trials after congruent shorter thantrials after incongruent	1,26	27	0	0.51
Congruency × Prev-Congruency	Smaller Simon effect after Incongruent than after Congruent	1,26	9.66	0.005	0.27
Time × Group × Congruency × Prev-Congruency^1^		1,26	4.69	0.03	0.15
Planned comparisons –	Sequential Gratton appeared only following MBIS-1*8 (time2)	1,11	6.62	0.025	0.38

^1^only at the MBIS groups. All other effects and interactions were not significant [i.e., (all Fs < 1)]. Supplementary material: See separate file.

For RT, the neutral condition was excluded for simplification purposes. Mean RTs and SDs are reported in Supplementary Table S2 in the Supplementary material. A main effect for time was found [*F*(*1*,*26*) *= 8*.*3*, *p < 0*.*008*, *η^2^ = 0*.*24*], such that the RTs in Time 2 were faster than the RTs in Time 1; a significant main effect was found for congruency [*F*(*1*,*26*) *= 25*.*3*, *p < 0*.*00003*, *η^2^ = 0*.*49*], such that RTs in the congruent trials were faster than the RTs in the incongruent trials-Simon effect; a significant interaction was found between group and congruency [*F*(*2*,*26*) *= 4*.*78*, *p < 0*.*017*, *η^2^ = 0*.*27*], such that the Simon effect appeared in the MBIS-2*4 and the control group beyond Time but not in the MBIS-1*8 group [*F*(*1*,*26*) *= 7*.*19*, *p < 0*.*05*, *η^2^ = 0*.*22*]; a significant interaction effect was found between time and congruency [*F*(*1*,*26*) *= 4*.*63 p < 0*.*04*, *η^2^ = 0*.*15*] such that the congruent trials were significantly reduced from Time 1 to Time 2 [*F*(*1*,*26*) *= 15*.*16 p < 0*.*0006*, *η^2^ = 0*.*36*] while incongruent trials showed only marginally significant reduction [*F*(*1*,*26*) *= 3*.*04 p < 0*.*09*, *η^2^ = 0*.*10*; see Table 3 and Supplementary Table S2].

Sequential analyses were also carried out. In an attempt to examine if the performance of the previous trial influences the performance of a following (current) trial, we conducted an ANOVA, which included a previous trial variable (previous congruent/previous incongruent). A significant main effect was found for the previous trial variable [*F*(*1*,*26*) *= 27*.*06 p < 0*.*00002*, *η^2^ = 0*.*51*] such that the RTs after congruent trials were shorter than the RTs after incongruent trials. Additionally, a significant interaction was found between congruency and previous trial congruency [*F*(*1*,*26*) *= 9*.*66 p < 0*.*004*, *η^2^ = 0*.*27*], such that a Gratton effect was found (see Table 3 and Supplementary Table S2). Namely, the Simon effect was smaller after incongruent trials than after congruent trials. This result contrasts previous findings regarding the Gratton effect in older adults, and indeed, does not reflect specific patterns of each group, as shown below.

**Figure 4:**
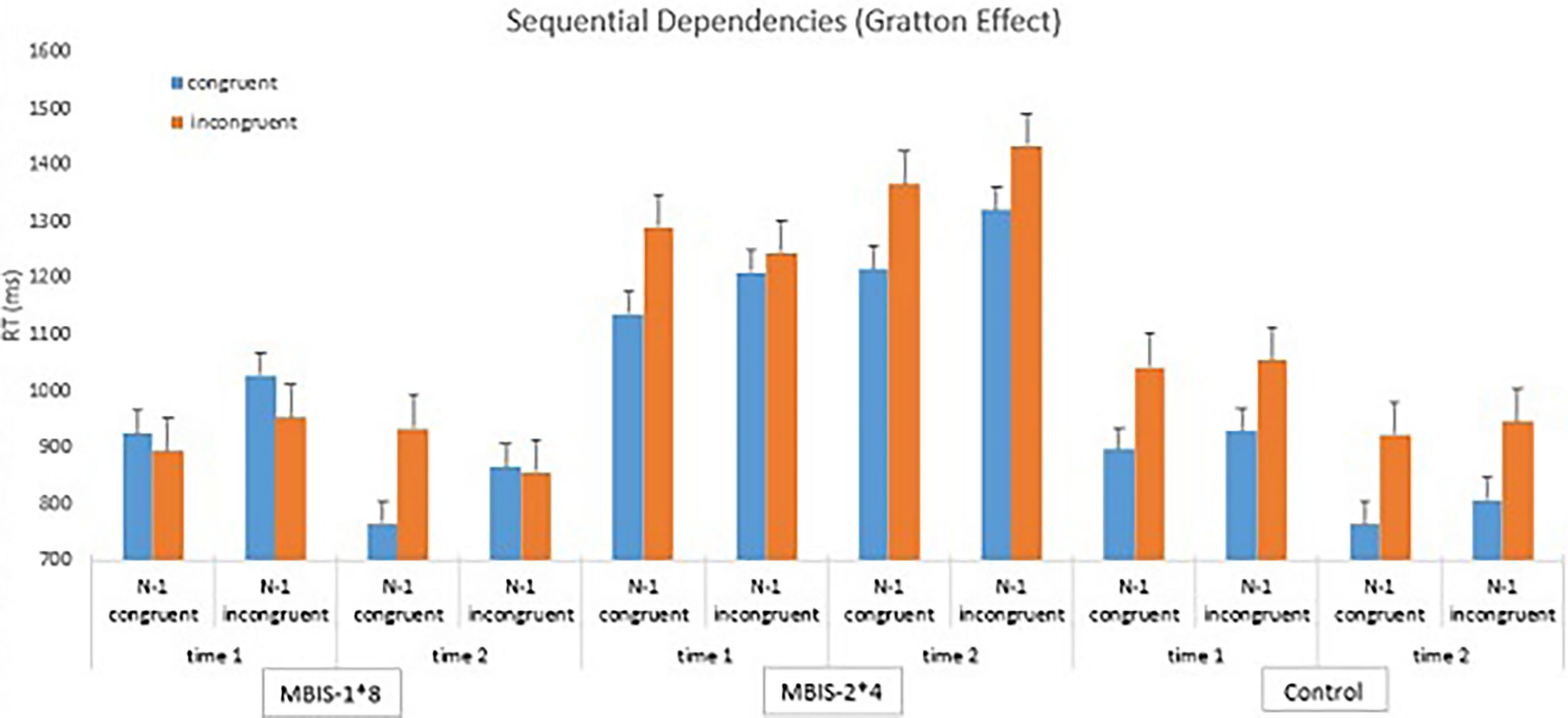
Averages and standard deviations in the sequential analyses for both groups before and after the intervention.

The four-way interaction between time, group, congruency, and previous trial congruency did not reach significance but yet had a medium effect size [*F*(*2*,*26*) *= 2*.*36*, *p < ns*, *η^2^ = 0*.*15*]. Following our hypothesis, we analyzed a four-way interaction looking only at the two MBIS groups. This four-way interaction was significant [*F*(*1*,*26*) *= 4*.*69*, *p < 0*.*04*, *η^2^ = 0*.*15*]. Further analysis showed that the Gratton effect appeared for the MBIS-1*8 group after the intervention [*F*(*1*,*11*) *= 6*.*62*, *p < 0*.*025*, *η^2^ = 0*.*38*], such that the Simon effect was significant after congruent trials but not after incongruent trials. Both the Control group and MBIS 4*2 groups showed the maladaptive, old adults’ typical pattern of sequential effects in time 2 (see Figure 4).

## Discussion

The aim of this study was to examine the influence of a brief mindfulness-based intervention appropriated for senior participants, on measures of emotional distress and cognitive control in healthy seniors. Another aim was to examine the influence of session frequency and intervention length on the amount of improvement in the aforementioned measures.

The main findings of this study were: The intervention, in both frequency forms, created a difference in reported levels of mindfulness, improved reported levels of depression and mood, and influenced cognitive functioning, compared to the control group, as will be detailed below.

### Mindfulness measures

In accordance with the research hypothesis, we found an improvement in the Non-reactivity to inner experience measure, whose score was the lowest prior to the intervention. This finding will be explained with the emotional and cognitive findings. In addition, the Non-judging of inner experience and Acting with awareness measures showed a decline after the intervention, while their scores prior to the intervention were the highest. This finding is surprising and would need to repeat itself in future research to be appropriately substantiated. In the context of this study, we can hypothesize that this decline is a result of the increase in attention that participants paid to their daily activities, as a result of the course. In other words, it is possible that the participants were “naïve” and thought of themselves (and reported accordingly) as being very aware prior to the intervention. Their enhanced awareness resulting from the intervention may have encouraged a more realistic reporting on acting with awareness and, subsequently, engendered a lower score in this measure. Along with enhanced awareness, Judging also increased toward “not aware enough behavior.’’ In other words, it is possible that the increase in Judging and the decrease in Acting with awareness actually reflect an improved and more accurate perception of personal experience among the senior participants.

No improvement was found for the measures Observation and Description; whose scores were the highest prior to the intervention. As in previous research (Morone et al., 2009), the base level of mindfulness skills was found to be higher in senior participants than it was in young participants. This makes it difficult to see improvement as a result of the intervention (i.e., ceiling effect).

### Emotional distress measures

The improvement in depression and mood measures in this preliminary pilot study points to the raw potential of brief mindfulness-based interventions for seniors in the community. This is even more impressive considering two elements: (1) Even though the base level of depression was not considered clinical, and the participant scores were low prior to the intervention, there was still an improvement in their state and their scores significantly declined; (2) This study examined a group of very older adults senior participants (the average age of the participants in this study was 82, higher than the average age of most MBI studies conducted on seniors), and the intervention still appeared appropriate for them and improved very important measures of the psychological wellbeing of this age group.

It is important to note that all the emotional measures changed in the same manner in both groups, irrelevant of session frequency and course length. This supports findings of Carmody and Baer (2009), which showed no correlation between course length and course results. However, it is possible that course length and session concentration had an “offsetting effect” on the results. On one hand, a lengthy intervention (MBIS-1*8) allowed for a more significant adaptation because of the amount of time the participants were involved in the course. On the other hand, it is possible that the 1-week intervals between sessions were too long of a break and interrupted the learning of a new skill, the opposite of which being an advantage of the second intervention (MBIS-2*4), in which two sessions a week were provided.

### Cognitive control

A difference was found between the groups’ performance of the Simon task, above and beyond time, such that the MBIS-2*4 group, which was characterized by fewer years of education and fewer social ties, had a greater Simon effect than the MBIS-1*8 group. Education and social ties act as resilience factors that decrease cognitive damage in old age (Stern, 2002), and it appears that they contributed to this gap in performance between the two groups.

Similar to previous research, the Simon effect in the senior participants of this study was greater than the Simon effect that was reported in the research literature on young participants. This finding suggests a difficulty in the inhibition response system in seniors (Aisenberg et al., 2014). In other words, senior participants display a weakened capacity to avoid information coming from a task-irrelevant dimension. In the task used in this study, the irrelevant dimension was the spatial location, and senior participants posed more difficulty avoiding this information than young participants.

Post the intervention, the Gratton effect appeared for the MBIS-1*8 group. This finding is difficult to explain considering the lack of a Simon effect prior to the intervention. However, its appearance suggests successful functioning, which is not common among seniors (Aisenberg et al., 2014), and appeared in this study only after the intervention. The difference between the groups in the Gratton effect can be due to the fact that the MBIS-1*8 group more significantly made use of what they learned in the course, due to their higher level of education and their improvement in the Nonreactivity to internal events measure. However, it is possible that the improvement in performance and the appearance of the Gratton effect are also due to the fact that this group participated in a lengthier course. In contrast to emotional changes that can be situational, cognitive processes mature over a longer period of time and are, therefore, expected to appear after a longer period of time (Chiesa et al., 2011). This can also explain the improvement in the emotional measures (i.e., depression and mood) and the lack of improvement in the main measure of cognitive control (Simon effect) in this study. It is important to note that the improvement in reaction time in the Simon task is expected due to repeated performance and, therefore, we will not assume that this achievement is a result of the interventions.

This study has several important limitations. First, most of our participants were older adults women, and generalization to older adults men needs further evidence. Second, the number of participants in this study is small and influenced the statistical power of the analyses, and the validity of the effectiveness conclusions. Many efforts were taken to encourage participation and to avoid attrition, such as personal reminders, make-up sessions, etc. Future research should maintain a small group of participants (as in this study) but attempt at increasing the statistical power *via* a larger number of groups. Third, due to the advanced age of the participants, it was difficult for some of the participants to follow instructions due to hearing loss, to concentrate during the meditation, and to read the written instructions for the home exercises. These are common difficulties in the senior population, and clearly affect the validity of this study, though as an ecological study it is part of the disadvantages. Another issue that limits the strengths of our results is related to differences between groups: First, because the participants were drafted from assisted living facilities, we distributed them into the different experimental conditions in a semi-randomized allocation (randomized between experimental and control but not for the type of experimental-frequency group). As a result, there were pre-intervention differences between the experimental groups in education and social ties, which possibly affected the results. Second, the frequency of sessions and length of course, which we wanted to examine, changed the number of practice days at home between the sessions. The groups had an equal amount of total-class time, but the MBIS-1*8 course required its participants to practice 7 consecutive days at home each week, while the MBIS-2*4 course required its participants to practice 2–4 days at home each week. As aforementioned, this likely influenced the learning process and the amount of dedication to the course. A third difference between the groups is the level of skillfulness of the course mentor. The two courses ended at the same time but began at different times. As a result, most of the MBIS-2*4 course sessions were offered after the MBIS-1*8 course sessions ended and, although the efforts to make the sessions identical across groups, the confidence and skillfulness of the mentor were definitely higher and the number of errors was fewer for the MBIS-2*4 group than they were for the MBIS-1*8 group. This was not reflected in the research findings, aside from *via* the amount of attrition in the MBIS-2*4 group, which was smaller. In addition, we compared experimental groups to a control group, which met weekly but with no structural program across sessions. Due to technical restrictions, not all questionnaires were administered in the control group, which further limits our comparison. Difficulties in measurement: First, due to time and location constraints, the Simon task was provided on different laptops, under different lighting conditions, and on different table heights, which damaged the reliability of the RT measurement in the cognitive task. These are expected constraints in the senior population, but still have an undeniable influence on the results. Second, the abbreviation of the intervention required choosing appropriate exercises that would reflect all the elements that contribute to the mindfulness intervention. However, it seems that for the senior population, repeated practice and high frequency are of great value. Thus, it makes sense to examine an appropriated course for seniors, which will combine advantages and will include bi-weekly sessions for a period of 8 weeks, and a total of 16 sessions.

Despite these limitations, the study’s strengths rely on its ecological nature. Older adults (aged 72–91) took part in this study, while maintaining their routine and sometimes unexpected life circumstances. Aging involves cognitive decline and emotional distress, and social interventions bear incredible preventative potential. Here, the social effect of the groups may have contributed to our emotional effects but was compared to a control group, who did not enjoy the same benefits.

We encourage future research to include a longitudinal measure after 6 months. This would enable us to understand how deep the aforementioned changes are. We also assume that the differences between groups will strengthen as a result of longitudinal influences. Likewise, it would be interesting to examine the association between participant traits and their experienced change as a result of the intervention. Various nursing home workers can be trained to conduct multiple courses of MBIS within the institutions.

In conclusion, a brief mindfulness-based intervention succeeded at engendering change in mindfulness measures, similar to the results reported from full MBSR interventions. Most importantly, the intervention succeeded in improving measures of psychological distress and cognitive capacity among senior participants. This was only a pilot study with a small sample size; hence caution should be made about generalizability to the larger population. Yet, it has great potential implications for the improvement of quality of life and psychological well-being of aging and aged individuals. In times of need, interventions that can be offered in a group setting, at a low price, and at different community centers or isolated nursing homes (as in a time of pandemic such as COVID-19 pandemic) are necessary. We provide an initial offer for a treatment-maintenance solution for seniors, with a ready-to-use protocol to administer immediately.

## Data availability statement

The original contributions presented in the study are included in the article/Supplementary material, further inquiries can be directed to the corresponding author.

## Ethics statement

The studies involving human participants were reviewed and approved by the ethic committee of the school of Social and Community Sciences at Ruppin Academic Center, R2017. The patients/participants provided their written informed consent to participate in this study.

## Author contributions

All authors agree to be accountable for the content of the work. MH performed the study as part of her MA thesis, wrote the protocol, ran the MBIS groups in nursing homes, analyzed the data, and wrote parts of the manuscript. DA-S supervised MHs’ work, in writing the protocol, analyzing the data, and writing the manuscript. All authors contributed to the article and approved the submitted version.

## Conflict of interest

The authors declare that the research was conducted in the absence of any commercial or financial relationships that could be construed as a potential conflict of interest.

## Publisher’s note

All claims expressed in this article are solely those of the authors and do not necessarily represent those of their affiliated organizations, or those of the publisher, the editors and the reviewers. Any product that may be evaluated in this article, or claim that may be made by its manufacturer, is not guaranteed or endorsed by the publisher.
