# Supplementary material for: Improving depressive symptoms and maintaining cognitive abilities of seniors within the nursing homes: A pilot study of brief mindfulness-based interventions for seniors in a semi-randomized trial
Source: Front Psychol. 2023 Aug 9;13:994336. doi: 10.3389/fpsyg.2022.994336 (PMC10446881; doi:10.3389/fpsyg.2022.994336)
Supplement: Supplementary file 1 [file Data_Sheet_1.docx]

Supplementary Material

**Table S1**

*Five Facet Mindfulness Questionnaire: Averages (SDs) and main effect for time*

|  |  |  | **Before Intervention (time 1) After Intervention (time 2) Main effect for Time** | | | | | | | |
| --- | --- | --- | --- | --- | --- | --- | --- | --- | --- | --- |
|  | **MBIS-1*8**  **(N=5)** | **MBIS-2*4**  **(N=10)** | | **All Participants**  **(N= 15)** | **MBIS-1*8**  **(N=5)** | **MBIS-2*4**  **(N=10)** | **All participants**  **(N= 15)** | **F** | **P<** | *η^2^_p_* |
| **Observing** | 3.4(.5) | 3.5(.8) | | 3.5(.7) | 3.8(.6) | 3.8(.5) | 3.8(.9) | <1 | ns |  |
| **Describing** | 4.2(.7) | 3.8(.7) | | 3.9(.7) | 4.1(2) | 3.7(1.1) | 3.9(.6) | <1 | ns |  |
| **Acting with awareness** | 5.0 (.7) | 4.1 (.8) | | 4.4 (.8) | 3.8(.6) | 3.2(1.3) | 3.4 (1.1) | 6.29 | .05 | .35 |
| **Nonjudging** | 4.4 (.7) | 4.2 (.9) | | 4.3 (.8) | 3.4(.5) | 3.3(.9) | 3.4 (.8) | 5.23 | .05 | .3 |
| **Nonreactivity** | 2.7 (.7) | 2.7 (.7) | | 2.7 (.6) | 3.8(.4) | 3.6(.5) | 3.7 (.5) | 5.29 | .05 | .31 |

**Table S2**

|  |  | **MBIS-1*8** | | | **MBIS-2*4** | | | **Control** | | | **All Participants** | | |
| --- | --- | --- | --- | --- | --- | --- | --- | --- | --- | --- | --- | --- | --- |
|  |  | **Before (Time1)** | **After (Time2)** | **Beyond Time** | **Before (Time1)** | **After (Time2)** | **Beyond Time** | **Before (Time1)** | **After (Time2)** | **Beyond Time** | **Before (Time1)** | **After (Time2)** | **Beyond Time** |
| **Congruency** | **Prev-Congruency** | 925(144) | 766(116) | 846(153) | 1136(374) | 1215(322) | 1173(353) | 896(238) | 763(257) | 830(256) | 973(395) | 888(328) | 931(315) |
|  | **Prev-Incongruency** | 1028(123) | 866(64) | 947(127) | 1290(475) | 1364(322) | 1325(415) | 932(220) | 815(277) | 873(257) | 1059(350) | 981(368) | 1021(359) |
|  | **Beyond Prev Trial** | 977(144) | 816(107) | 896(150) | 1213(435) | 1290(334) | 1249(392) | 913(230) | 788(268) | 851(258) | 1015(326) | 934(349) | 975(340) |
| **Incongruency** | **Prev-Congruency** | 894(86) | 934(156) | 914(128) | 1210(346) | 1319(410) | 1261(392) | 1033(322) | 909(263) | 971(300) | 1062(322) | 1023(348) | 1043(336) |
|  | **Prev-Incongruency** | 952(100) | 855(157) | 904(141) | 1244(419) | 1433(474) | 1332(456) | 1053(264) | 945(301) | 999(289) | 1094(320) | 1064(411) | 1079(368) |
|  | **Beyond Prev Trial** | 923(98) | 895(162) | 909(134) | 1227(384) | 1376(449) | 1297(423) | 1043(295) | 927(282) | 985(295) | 1078(325) | 1043(369) | 1061(349) |
| **Beyond Congruency** | **Prev-Congruency** | 909(120) | 850(161) | 880(145) | 1173(362) | 1267(375) | 1217(371) | 967(292) | 839270) | 903(289) | 1018(312) | 957(345) | 988(330) |
|  | **Prev-Incongruency** | 990(118) | 861(120) | 925(136) | 1267(440) | 1398(409) | 1329(436) | 995(252) | 882(296) | 938(281) | 1077(336) | 1023(390) | 1050(365) |
|  | **Beyond Prev Trial** | 950(126) | 855(142) | 903(142) | 1220410) | 1333(398) | 1273408) | 980(274) | 860(284) | 920(285) | 1047(325) | 989(369) | 1019(349) |

*Averages (SDs), for RTs (RT in ms) in the Simon task.*
